# Do Biliary Salts Have Role on Acute Kidney Injury Development?

**DOI:** 10.14740/jocmr2261w

**Published:** 2015-07-24

**Authors:** Thiago Gomes Romano, Jose Mauro Vieira Junior

**Affiliations:** a Nephrology Department, ABC Medical School, Hospital Sirio Libanes, Brazil; b Intensive Care Unit, Hospital Sirio Libanes, Brazil

**Keywords:** Bile cast, Acute kidney injury, Nephropathy

## Abstract

Acute kidney injury (AKI) is a major complication in patients with acute liver failure and chronic liver disease. Hemodynamic changes appear to be the principal alterations in these conditions, therefore there should be no known structural abnormalities responsible for AKI. On the other hand, several authors have published data on structural changes known as bile cast nephropathy or cholemic nephrosis, which basically consist of the presence of bile casts in tubular lumen analogous to those observed in myeloma. Although these findings are well documented, there is a lack of reproducibility by other authors. This paper aims to discuss, through evidence-based medical literature, the role of biliary salts on kidney injury development.

## Introduction

Acute kidney injury (AKI) is a major complication in patients with acute liver failure and chronic liver disease. Hemodynamic changes seem to be the main alterations in these scenarios, a condition named hepatorenal syndrome (HRS).

The incidence of HRS, defined as worsening of serum creatinine to levels above 1.5 mg/dL within 2 weeks for type 1 HRS and over several months for type 2 HRS, is not well established but can occur in up to 40% of cirrhotic patients [1].

The pathophysiology of HRS is mainly due to intrarenal vasoconstriction which results in a functional impairment of the kidneys. Sympathetic activation, angiotensin II, endothelin and nitric oxide have been described as potential pathogenetic mechanisms which account for the systemic and renal hemodynamic disturbances. Therefore there should be no known or well described structural abnormalities that contribute to renal dysfunction [2].

On the other hand, several authors have published data on structural changes named as bile cast nephropathy or cholemic nephrosis, which basically consist of the presence of bile casts in the tubular lumen analogous to those observed in myeloma or myoglobin casts [3-6]. This entity was described in early 1900, but for some reason has rarely been investigated or cited in the latest decades. Recently, some authors have rekindled the subject, shedding light upon the conceivable role of bile casts or biliary salts on renal injury development.

Although the findings cited above are well documented, there is a lack of reproducibility by other authors. Moreover, there are also many data, mainly from experimental studies, of the potential protective role of bilirubin on oxidative stress of the kidneys and its role on kidney function protection.

This paper aims to discuss, through evidence-based medical literature, the role of biliary salts on kidney injury development.

## Evidence of Biliary Salt-Induced AKI

The concept that biliary salts may induce AKI is not new, Haessler et al in 1922 [7] analyzed the urine of humans and dogs with jaundice and concluded that biliary salts could be found in urine sediment and further studies showed that, as the liver injury resolves and renal function recovers, the biliary salt tends to disappear. However in the last decade only few cases reports addressed this issue [8].

Recently, van Slambrouck et al in a clinicopathologic study with 44 patients with jaundice submitted to histological analysis (41 autopsies and three renal biopsies) demonstrated that 24 patients had bile casts with involvement of distal nephron and in six of them with extension to proximal tubules. The mean total bilirubin levels in the group with bile cast nephropathy was 26.2 mg/dL against 15.1 mg/dL in the control group (P = -0.001). Acute tubular injury (ATI) was also observed in 77% of the cases in the bile cast nephropathy group, characterized by tubular epithelium with attenuated cytoplasm or loss of proximal tubular brush borders [9]. Van Slambrouck et al concluded that bile cast nephropathy is a histological proven entity, with more than half of the patients prone to bile deposition in renal tubules.

Bal et al also observed bile cast nephropathy in three post-mortem kidney biopsies from patients who died from subacute hepatic failure [10], similar to the findings of Shet et al, where bile cast was more extensive in biliary cirrhosis involving four of seven children with extrahepatic biliary atresia [11].

The fact that bilirubin may play a role in the development of AKI in hepatic failure can explain why terlipressin was effective in only 13% of patients with serum bilirubin > 10 mg/dL (171 μmol/L) compared with 67% of patients with lower serum bilirubin levels according to Nazar et al [12].

The potential role of renal excretion of biliary salts as a causative factor for AKI was also suggested by Fickert et al in an animal model where a 3-day common bile duct ligation (BDL) induced renal tubular epithelial injury predominantly at the level of aquaporin 2-positive collecting duct with tubular epithelial and basement membrane defects. Receptors (para que? Knockout para AQP? Ou receptor de VR2?) knockout mice (with a hydrophilic biliary salt pool) were completely protected from AKI [13].

The mechanism by which bilirubin may be toxic to the kidneys seems to be also inflammatory, besides obstructive, as described by Ozturk et al. Through administration of sirolimus in animals submitted to BDL, they demonstrated that the drug decreased tubulointerstitial inflammation in the kidney induced by BDL [14]. As a consequence, sirolimus provided (or led to a protection) a protection effect against the BDL-induced AKI.

Hemodynamic changes could also have a role in AKI induced by biliary salts, as shown by Paddillo et al in another experimental study where obstructive jaundice has the ability to increase renin and aldosterone levels. As a result, renal blood flow in this situation is compromised [15]. That study strengthened the idea that the main mechanism of AKI is indeed functional but with a role of biliary salts underlying (behind) these alterations.

Bilirubin toxicity to the kidneys, hitherto and with the cumulative evidence, would be considered a multifactorial entity, where tubular obstruction, tubulointerstitial inflammation and renal hemodynamics are the potential main mechanisms described.

## Bilirubin and Its Protective Effect on Kidney Function

On the other hand, in contrast to the data demonstrated above, endogenous bilirubin is an established cytoprotectant and antioxidant [16, 17]. In experimental studies, it has been shown that exogenous bilirubin is protective against oxidative stress after ischemia-reperfusion injury in the kidney [18]. Aqui tem mais uma do mesmo grupo = *in vivo*, com protecao parcial apenas = Kirkby et al, AJP 2006 (te mandei).

Also higher bilirubin levels are associated with low prevalence of death, coronary artery disease [19] and cancer mortality [20]. Deetman et al in a prospective study with renal transplant recipients have shown that higher bilirubin levels were independently associated with lower levels of late graft failure [21].

Similar findings were published by Oh et al, where the administration of exogenous bilirubin protected against cyclosporine-induced nephropathy in rats. The mechanism of protection in that study seemed to result from amelioration of tubulointerstitial fibrosis and tubular apoptosis [22].

Pereira et al, in an interesting study with male rats with hepatic fibrosis induced by ligation of common bile duct, demonstrated that, after 6 weeks, rats with ligation of the bile common duct showed features of HRS including increase in serum creatinine in contrast to the sham group. The histological findings in the first group were consistent with the classical medical literature findings of no structural changes in the kidneys [23]. The data from this study reinforce our current understanding that HRS is functional in nature and occurs as a consequence of hemodynamic changes associated with portal hypertension, despite bilirubin elevations.

Ligation of the common bile duct has also shown to confer resistance to glycerol-induced acute tubular necrosis in rats, as published by Leung et al. This protection might be due to induction of heme-oxygenase-1, a cytoprotective molecule, in the kidney and liver [24]. Bilirubin is one of the main products of heme oxygenase; when induced in stress conditions, tissue heme oxygenase expression confers antioxidant and cytoprotective effects, mediated probably by its downstream products of degradation, such as bilirubin [25, 26].

Heme oxygenase-1 role on kidney protection was investigated by Guo et al. In an experimental study, cirrhotic rats were divided into three treatment groups: zinc protoporphyrin group, cobalto protoporphyrin group and sham group. Biliary cirrhosis was induced by BDL in all groups. Zinc and cobalto group received intraperitoneal injection of those substances 24 h before blood sample collections and heme oxygenase-1 expression was detected by reverse-transcription polymerase chain reaction. Independently of the intervention group serum creatinine levels were lower in cirrhotic rats with higher heme oxygenase-1 expression.

The strongest evidence of biliary salt-induced AKI is the histological finding of bile cast nephropathy, but this can merely reflect reduced glomerular filtration rate (GFR), through a reduction in the washout of the casts, as stated by Heyman et al in a commentary of van Slambrouck’s paper. In the same letter the authors cited the potent endogenous antioxidant role of bilirubin in experimental settings [27]. Rats subjected to BDL with the development of biliary cirrhosis were more resistant to contrast nephropathy and their medullary thick limbs hardly developed hypoxic injury [28].

Fajers studied four groups of rabbits, divided into, cholemia, or no cholemia with or without renal ischemia. They found insignificant morphologic changes that could be explained solely by the ischemia alone, although cholemia duration was only 2 days [29].

Thus, the findings in literature are very controversial on the role of bilirubin on kidney injury development. Table 1 [9, 10, 13, 15, 23] and Table 2 [21, 22, 24, 30] summarize some of the studies that had addressed these issues until the current date.

**Table 1:** Summary of the Main Studies With Evidence of Pro-Toxic Effects of Bilirubin in the Kidneys

Authors/year	Methods	Results	Conclusion
Padillo et al, 2009 [15]	Experimental study. Animals submitted to common bile duct ligation had their renin, aldosterone, endothelin-1 and prostaglandine E2 studied and compared to control group.	Both groups had the same values for diuresis, renin and creatinine clearance at 24 h. Animals with obstructive jaundice had lower sodium concentration and an increase in aldosterone levels (P < 0.03), endothelin-1 (P < 0.001) and prostaglandine E2 (P < 0.001) in urine.	Vasoactive hormones may play a role in renal complications during obstructive jaundice.
Fickert et al, 2013 [13]	Experimental study. Animals submitted to 3-day common bile duct ligation, with a group of receptor knockout (para que?) mice for bile acids and (?) renal histology analyzed.	Bile common duct ligation induced renal tubular epithelial injury predominantly at the level of collecting ducts, followed by progressive interstitial nephritis and tubulointerstitial fibrosis. Knockout mices were completely protected from renal fibrosis.	Urinary excretion of bile acids represents a trigger for renal tubular epithelial injury leading to cholemic nephropathy.
Van Slambrouck et al 2013 [9]	Clinicopathologic study of 44 subjects (41 autopsies and three biopsies) from jaundice patients.	24 patients had bile casts with involvement of distal nephron tubules for?? six severe cases. Eleven of 13 patients with hepatorenal syndrome and all 10 with cirrhosis had tubular bile casts.	Bile cast nephropathy is an appropriate term for severe form of renal injury observed in cirrhotic patients.
Pereira et al, 2008 [23] Esse parece mais table 2	Experimental study. Male Wistar rats were submitted to sham surgery or bile duct ligation. Determination of renal function and histology samples were obtained after 6 weeks.	At 6 weeks the group with bile duct ligation showed features of hepatorenal syndrome including increase in serum creatinine and reduction of creatinine clearance, water excretion and urinary sodium concentration. Histological analysis has shown no alterations.	Bile duct ligation produced progressive renal dysfunction, although without structural changes in the kidneys, characterizing functional HRS.
Bal et al, 2000 [10]	Post-mortem histological analysis from patients died from subacute hepatic failure.	Bile cast nephropathy was observed in three of the patients.	Bile cast nephropathy is an important finding in the kidneys of icteric patients.

**Table 2:** Summary of the Main Studies With Evidence Against Toxic Effects of Bilirubin in the Kidneys

Authors/year	Methods	Results	Conclusion
Leung et al, 2001 [24]	Experimental study. Animals were submitted to common bile duct ligation and hypertonic glycerol was used to induce acute tubular necrosis (ATN). Renal injury was assessed by plasma creatinine concentration and histology.	Ligation of the common bile duct markedly reduced acute renal injury evidenced by less severe ATN and lower plasma creatinine. Ligation of the bile duct induced heme oxygenase-1 expression in the kidneys.	Ligation of bile common duct confers resistance to glycerol-induced acute renal injury which may be related to the expression of heme oxygenase-1 in the kidneys.
Guo et al, 2011 [30]	Experimental study. Animals divided into groups with biliary cirrhosis induced by bile duct ligation and sham. Expression of heme oxygenase-1 in kidneys was analyzed as serum creatinine and renal blood flow.	Heme oxygenase-1 expression, serum creatinine levels and renal blood flow were lower in the cirrhotic group (P < 0.05).	Intervention to increase the expression of heme oxygenase-1 in kidneys played a role in bilirubin protective effect in renal failure.
Deetman et al, 2012 [21]	Prospective data collected from August 2001 and July 2003 from non-icteric renal transplant recipients patients with a functioning graft for > 1 year.	Median data follow up to 7.1 years. Circulating levels of bilirubin were inversely associated with late graft failure, independently of urinary protein excretion, calcineurin inhibitors and gender.	Findings consistent with a protective effect of increased endogenous bilirubin against development of late graft failure in renal transplant recipients.
Oh et al, 2013 [22]	Experimental study. Male rats were submitted to intraperitoneal injection of bilirubin three times daily for 1 week before the administration of ciclosporine and a control group only with ciclosporine administration.	Ciclosporine induced increase in urine kidney injury molecule-1 (KIM-1) and neutrophil gelatinase-associated lipocalin (NGAL). Bilirubin reduced KIM-1 (P < 0.05) while NGAL exhibited a downregulation trend. The protein expression of NOX4 and p22phox was reduced by bilirubin and also apoptosis evaluated by TUNEL assay was ameliorated by bilirubin injection (P < 0.01).	The direct administration of bilirubin protected against ciclosporine induced tubular injury via inhibition of oxidative stress and apoptosis.

## Questions to Be Answered Before the Announcement of a New Entity

We think that some points deserve to be considered, in order to account for the differences in the results in the past studies, which could account for the confusion in the literature and even explain the apparent lack of interest on the subject along the latest decades. 1) In order to identify bile cast within the tubules, the kidney specimens should receive a specific histochemical stain (Hall stain). Even using specific stains, not all icteric patients will have deposit. For instance, only 42% of icteric patients without HRS had bile casts tubular deposit in the van Slambrouck et al’s study. Differences in the specimens processing could account for the disagreement among the findings. 2) The possible explanations of the lack of agreement between bile deposit and the development of AKI: bilirubin might conceivably be only an innocent bystander, although it is unlikely; bilirubin may indeed have deleterious consequences only in certain models of disease; or bilirubin might carry a risk to injury, depending on a concurrent ischemia, reduced GFR, another associated local tubular condition such as lower urinary pH, and the duration of the deposit or its extension. 3) Last, as already cited, the deposit might result merely from the reduced GFR, being as marker of decreased GFR.

## Conclusion

AKI in the context of liver disease is mainly described as a hemodynamic phenomenon but few data in medical literature shed light into an obstructive role of bilirubin mainly in distal tubule. Another interesting point is the potential pro-inflammatory effect of biliary salts and its role in terlipressin resistance in the treatment of HRS.

On the other hand, there is a large body of evidence that bilirubin is an antioxidant and a kidney protective substance through reduction of tubular apoptosis and tubulointerstitial inflammation, although this fact has yet to be confirmed on clinical grounds.

The lack of agreement in medical literature may be due to methodological problems or due to misinterpretation of the data, where bile cast nephropathy might be only an epiphenomenon secondary to a GFR reduction in cirrhotic patients.
